# Pharmacists’ background, interests, barriers, self-perceived competence and confidence to design and undertake pharmacy practice-based research in the GCC geographic area

**DOI:** 10.1186/s12909-020-02346-4

**Published:** 2020-11-07

**Authors:** Farah Kais Alhomoud

**Affiliations:** Department of Pharmacy Practice, College of Clinical Pharmacy, Imam Abdulrahman Bin Faisal University, Dammam, Saudi Arabia

**Keywords:** Pharmacists, Clinical competence, Research confidence, Pharmacy research

## Abstract

**Background:**

The absence of ability and certainty to design and undertake pharmacy practice-based research (PPBR) was a major issue among pharmacists worldwide as reported in previous literature, despite them having an interest in conducting PPBR. Therefore, this study aimed at highlighting the research background of the Gulf Cooperation Council (GCC) pharmacists which are the six Arab states of the Arabian Gulf, and examining barriers to conducting PPBR. In addition, to determine the self-perceived level of competence and confidence when planning and conducting PPBR.

**Methods:**

This is a descriptive, cross-sectional questionnaire-based study, performed among pharmacists working in the GCC countries (i.e. Saudi Arabia, United Arab Emirates, Kuwait, Bahrain, Oman and Qatar). A pre-validated questionnaire was distributed to a convenience sample, via distribution of 500 research participation cards to conference attendees. These cards a quick response (QR) code, which should be scanned via mobile phone, to direct all readers to the online survey. All pharmacist delegates attending the conference (i.e. the Saudi International Pharmaceutical Sciences Annual National Conference (SIPHA) and Dubai International Pharmaceuticals and Technologies Conference and Exhibition (DUPHAT) in 2020 and who are working in one of the GCC co.

untries as pharmacists were considered. The data were analysed using the Statistical Package for the Social Sciences (SPSS) and Excel software.

**Results:**

Two hundred and fourteen pharmacists were included. Seventy percent of the pharmacists had past PPBR background. Confidence and competence of pharmacists for research skills in regard to employing appropriate inferential statistical test, choosing software for statistical analysis, drafting a comprehensive plan for data analyses and determining appropriate sample size were weak. Highest competence and confidence were seen in preparing a presentation and searching the literature. Pharmacists with previous research experience (K-W: *p* = 0.001) and training (K-W: *p* = 0.003) had an overall ability to conduct PPBR. In addition, they had more interest in conducting PPBR (MWU = 3061.500, z = − 4.126, *p* = 0.000) and in learning about how to do so (MWU = 8698, z = − 1.898, *p* = 0.050).

**Conclusions:**

Pharmacists practicing in the GCC geographic area realized the importance of planning and conducting PPBR and were more confident and competent to undertake and contribute to PPBR, except for skills related to statistical analysis. Therefore, training programmes especially for biostatistics and data analysis are mandatory to enhance pharmacists’ research capabilities.

## Background

Pharmacy practice-based research (PPBR) is defined as “research that attempts to inform and enhance pharmacists’ understanding of the way practice should focus to ensure informed medicine information, as well as guarantee evidence-based practice” [1]. Pharmacy practice research should be frequently performed to investigate the necessity, efficacy and efficiency of all pharmacy services performed in different practice settings aiming to provide quality care, optimize medicine use and reduce medication errors for individuals [2, 3]. Especially that previous evidence support the role of pharmacists in providing quality health services in different settings as well as to their noticeable abilities to start and maintain a partnership with their patients and healthcare providers [4, 5]. Therefore, conducting such research will provide a guide and evidence to inform best practices [5]. The World Health Organisation (WHO) and the International Pharmaceutical Federation (FIP) initiated the ‘nine star pharmacist’ concept, which acknowledged that a well-rounded pharmacist should have the following criteria: a good decision maker, communicator and manager with leadership characteristics, entrepreneur, constant learner, and have teaching skills and research abilities [6]. Therefore, in addition to providing support and participating in PPBR, pharmacists are also required to undertake PPBR to generate a novel knowledge and integrate its results into patient care practice [7, 8]. However, this can be challenging because, although pharmacists have recognized the importance of PPBR and are interested in participating [2, 5, 9], this does not translate into a capability to undertake research. Worldwide, previous literature has reported several barriers to pharmacists’ participation and conduction of PPBR, such as: inadequate knowledge of research design [7, 10, 11], lack of time and job support [7, 11–13], lack of research experience [9, 13], inadequate knowledge, training and financial funds [2, 5], the absence of education curricular and postgraduate training and mentorship [8], and, finally, low publication rate [5, 7, 14]. However, none of the previous studies have looked at the barriers to designing and conducting PPBR in the GCC countries. Addressing these barriers will enable us to have a clear picture on the nature and size of this problem in the Gulf countries to make recommendations and suggest some suitable interventions to overcome these barriers. Therefore, this study aimed at highlighting the research background of the GCC pharmacists, estimating their publication rate and determining the barriers to conducting PPBR; seeing whether they have an interest in attending a postgraduate education programme to empower their research capabilities or not; and determining the self-perceived level of competence and confidence when planning and conducting PPBR among pharmacists in GCC countries all together and separately.

## Methods

Study setting, recruitment, data collection and the study instrument:

### Study design

This is a descriptive, cross-sectional questionnaire-based research, performed among pharmacists who work in the GCC countries. The study was conducted during the Saudi International Pharmaceutical Sciences Annual National Conference (SIPHA) in January 2020 and Dubai International Pharmaceuticals and Technologies Conference and Exhibition (DUPHAT) in February 2020.

### Data collection instrument

A pre-validated questionnaire was adopted to better fit the purpose of this study [15]. The corresponding author received the permission to use the questionnaire. The questionnaire was distributed in the English language only and was divided into six parts: participants’ characteristics, research background and interest, barriers to participation, self-assessment of competence, and self-assessment of confidence to conduct research, and postgraduate training interest and preference. The questionnaire showed a moderate strong reliability (Cronbach’s alpha = 0.60) and acceptable content validity [15]. Two experienced pharmacy practice academics read and evaluated the questionnaire for face and content validity. Based on their views and feedback, the questionnaire was seen to be suitable and no modification was performed.

### Study population and data collection

A convenience sampling method was employed to ensure the maximum participation for an online-based multi-country survey study. The questionnaire was distributed during the Saudi International Pharmaceutical Sciences Meeting and Workshops (SIPHA) in January 2020 and Dubai International Pharmaceuticals and Technologies Conference and Exhibition (DUPHAT) in February 2020. These conferences were chosen because they are the two largest pharmacy conferences in the Gulf area. More than 27,500 visitors and participants attended the conferences [16]. They are organized annually to bring experts and those interested in the field of pharmacy. They bring pharmacists and researchers together for sharing knowledge and improving health-related practice. In addition, they provide a variety of lectures, new research projects presented as posters or presentations from pharmacy researchers and leaders to gain valuable knowledge and help pharmacists with their continuous learning and growth in their desired field. A total of 500 research participation cards were distributed among conference attendees. These cards include an introductory paragraph about the title, aim and the eligibility criteria for all participants to participate in this study, in addition to a quick response (QR) code, which should be scanned via mobile phone, to direct all readers to the online survey. The first page of the online survey contained an information sheet giving potential participants the necessary understanding of the purpose of the study and the inclusion criteria. Participants were asked to read the information carefully and, before starting to complete the survey, they had to sign a consent form agreeing that they had read and understood all the information presented and that they agreed to take part.

#### Sample size

Sample size was calculated using a web-based sample size calculator [17]. Considering 5% two-tailed alpha error (type one error for α value = 0.05), 95% confidence interval and 7% precision rate, the required sample size was 196. Adjustment was carried out for missing/non-response error at 10% and added to the sample size. Finally, the required sample size was 218 pharmacists. Post hoc power calculation was performed and reported more than 90% power was achieved for the sample size [18].

#### Data processing and analysis

A total of 214 participant were analysed using the Statistical Package for Social Scientists (IBM SPSS version 24.0) and Excel software. After cleaning the missing data and incomplete information, variables were coded and recoded as needed. Descriptive statistics were used to describe the background characteristics of the study variables and items. Frequencies (N) with their corresponding percentages (%) were reported. Confidence and competence level of pharmacists’ self-assessment was compared among GCCs using Chi-square/Fisher exact test (where it was applicable), to indicate which research skills pharmacists were most and least confident and competent to do, for all GCC countries together and separately. *P*-values lower than 0.05 were considered statistically significant. Associations between participants’ previous research experience and training and their views on their overall ability to design and conduct PPBR was evaluated using Kruskal-Wallis test (K-W). In addition, Mann-Whitney-U (MWU) test was carried out in order to make a comparison of the relation between pharmacists who had past research experience and those who did not have and their interest in conducting or learning about how to conduct PPBR.

## Results

### Response rate

A total of 500 research participation cards were distributed among conference attendees to take part in this study. Of those who met the criteria and agreed to take part, 214 filled out and completed the questionnaire (response rate 42.8%).

Characteristics of the sample:

Pharmacists ranged in age from 21 to 59. There were more than twice as many female participants as male ones (Male = 63: 29% and Female = 151: 71%). With regard to the distribution of pharmacists from GCC countries, they were mostly from the Kingdom of Saudi Arabia (KSA) (*n* = 90: 42%), followed by Kuwait and Oman (*n* = 40: 19%), and then the United Arab Emirates (UAE) (*n* = 30: 14%), Bahrain (*n* = 11: 5%) and finally Qatar (*n* = 3: 1%). More than half of the participants (*n* = 130: 61%) held a bachelor’s degree in Pharmacy (B.Pharm) (*n* = 106: 50%) or were a Doctor of Pharmacy (PharmD) (*n* = 24: 11%). Almost two-thirds of the sample (*n* = 134: 63%) were hospital pharmacy employees, followed by retail/community pharmacists (*n* = 37: 17%), and academics and researchers (*n* = 34: 16%). More than one-third of the sample (*n* = 73: 34%) spent 1–5 years in the field of pharmacy practice, followed by 6–10 years (*n* = 55: 26%) (see Table 1). There was a statistical significant difference between pharmacists with higher education and lower education (*p* = 0.01). The higher the education is, the more the pharmacists were interested in conducting research and the more they were able to design and conduct health-related research (*p* = 0.001). Whilst, no statistically significant difference was observed between those with B-Pharm’s vs PharmD degrees or those practicing in academia or other fields of pharmacy with all other diameters.

**Table 1:** Frequency distribution for GCC pharmacist demographics (*n* = 214)

Variables	Group	Frequency (n)	Percentage (%)
Age	21–30	88	41.0
	31–40	96	45.0
	> 40	30	14.0
Gender	Female	151	71.0
	Male	63	29.0
Country of residence	KSA	90	42.0
	Kuwait	40	19.0
	Oman	40	19.0
	UAE	30	14.0
	Bahrain	11	5.0
	Qatar	3	1.0
Marital status	Married	136	64.0
	Single	78	36.0
Highest degree achieved	Bachelor of pharmacy (Bpharm)	106	50.0
	Doctor of Pharmacy (PharmD)	24	11.0
	Master’s Degree	64	30.0
	Doctor of Philosophy (PhD)	20	9.0
Place of obtaining first professional pharmacy degree	University from home country	148	69.0
	From a foreign country	66	31.0
Field of pharmacy practice in	Hospital pharmacy	134	63.0
	Retail or community pharmacy	37	17.0
	Academia and research center	34	16.0
	Others (Administration, pharmaceutical company & Industry)	9	4.0
**The number of years spent in the field of pharmacy practice**	< 1 year	32	15.0
	1–5 years	73	34.0
	6–10 years	55	26.0
	11–15 years	22	10.0
	> 15 years	32	15.0

### Research background and interests

Over two-thirds of the sample (*n* = 150: 70%) had research practice experience, which was obtained either by training through attending workshops (*n* = 95: 45%) or seminars (*n* = 58: 27%), during undergraduate or postgraduate study, or during their job. In contrast, just under one-third of the sample (*n* = 64: 30%) had no research experience and declared that they had not received any research training (*n* = 61: 28%). Fifty-one percent of participants had not participated in any specialized short courses, whereas almost one-third of the sample (*n* = 74: 35%) had 1–6 months’ experience of specialized short courses, whereas the rest (thirty participants; 14%) had > 6 months’ experience. More than three-quarters of the participants (*n* = 164: 77%) had an interest in conducting health-related research and were interested in learning about how to do so (*n* = 170: 79%).

When participants were asked to assess their overall ability to design and conduct PPBR, more than half of the sample (*n* = 131: 61%) claimed that their abilities were good to excellent. In contrast, more than one-third (*n* = 83: 39%) felt they had fair to poor ability. In addition, more than half of the sample (*n* = 114: 52%) stated that they had sometimes (*n* = 77: 36%) or had never been involved (*n* = 34: 16%) in research as participant, or as a principal investigator or co-investigator (59%).When participants were asked about the number of peer-reviewed journal articles published, or posters and/or abstracts presented within the last 5 years, more than half of the sample revealed that they had never published an article in a peer-reviewed journal (*n* = 123: 57%), or presented a poster/abstract (*n* = 107: 50%) at a local/regional conference in the last 5 years (see Table 2). There was a significant difference between participants who had past research experience (K-W: *p* = 0.001) and previous research training (K-W: *p* = 0.003) and their views on their overall ability to design and conduct PPBR. In addition, there was a significant difference between participants with or without previous research experience regarding their interest to conduct PPBR (MWU = 3061.500, z = − 4.126, *p* = 0.000) and learn about how to conduct PPBR (MWU = 8698, z = − 1.898, *p* = 0.050). Therefore, pharmacists with previous research experience were more likely to acknowledge their interest to conduct and learn how to conduct PPBR.

**Table 2:** Frequency distribution for GCC pharmacist research background and research interests variables (*n* = 214)

Variables	Group	Frequency (n)	Percentage (%)
Have any previous research experience?	No	64	30.0
	Yes	150	70.0
Previous research related training during undergraduate, postgraduate or during job	No training obtained	61	28.0
	Obtained training:	153	72.0
	Workshop	95	45.0
	Seminar	58	27.0
Experience on specialized short course	No	110	51.0
	Yes	104	49.0
	1–6 months	74	35.0
	7–12 months	17	8.0
	12 month	13	6.0
Do you have interest in conducting health-related research?	Not interested	50	23.0
	Interested	164	77
Are you interested in learning about how to conduct health-related research?	Not interested	44	21.0
	Interested	170	79.0
Overall ability to design and conduct health-related research	Poor	28	13.0
	Fair	55	26.0
	Good	101	47.0
	Excellent	30	14.0
Involvement in research as a subject or a respondent	Never	34	16.0
	Sometimes	77	36.0
	Often	48	22.0
	Usually	32	15.0
	Always	23	11.0
Involvement in research as a principal investigator or co-investigator	Never	64	30.0
	Sometimes	62	29.0
	Often	34	16.0
	Usually	36	17.0
	Always	18	8.0
Number of peer-reviewed journal articles published within the last 5 years	0 (No)	123	57.0
	1–3	70	33.0
	≥4	21	10.0
Number of peer-reviewed posters and/or abstracts in local/regional conference since last 5 years	0 (No)	107	50.0
	1–3	79	37.0
	≥4	28	13.0

### Barriers and interest to conducting research

When participants were asked about their barrier to conducting PPBR, the majority (*n* = 188: 88%) revealed that they had experienced a barrier such as lack of the following: time (*n* = 123: 57%), job support (*n* = 112: 52%), funds (*n* = 79: 37%), adequate knowledge (*n* = 71: 33%), self-esteem (*n* = 27: 13%), and interest (*n* = 17: 8%).

The majority of participants (*n* = 186: 87%) were interested in postgraduate studies including Ph.D. (*n* = 76: 36%), masters (*n* = 64: 30%) and residency and/or fellowship (*n* = 46: 21%). The most three reported areas of interest in pharmaceutical sciences among those participants were clinical and pharmacy practice (*n* = 145: 68%), pharmaceutics (*n* = 19: 9%) and pharmacogenomics (*n* = 18: 8%), whereas the least reported areas of interest were medicinal chemistry (*n* = 15: 7%), pharmacognosy (*n* = 9: 4%), pharmacokinetics (*n* = 8: 4%) and pharmacology (*n* = 0: 0%).

### Self-perceived competence and confidence of GCC pharmacists when planning and conducting PPBR

Participants (*n* = 214) were asked to choose from 3-point Likert-scale response options to assess their competence and confidence to conduct PPBR (i.e. [confident −1, moderately confident −2, not confident −3, and competent − 1, moderately competent - 2, and not competent − 3]). The most reported research domains by participants in regard to being competent when conducting PPBR were in preparing a presentation (*n* = 126: 59.7%), searching the literature (*n* = 108: 50.5%) and ethical considerations (*n* = 102: 47.7%). In contrast, participants did not select and employ proper “INFERENTIAL” statistical tests (*n* = 99: 46.9%), and were also found to be not competent in statistical analyses using software (e.g. STATA, SPSS, EpiInfo) (*n* = 93: 44.1%) and drafting a comprehensive plan for data analyses (*n* = 72: 34.1%). Similarly, participants were seen to be more confident in preparing a presentation (*n* = 118: 55.9%), searching the literature efficiently (*n* = 109: 51.7%) and ethical considerations (*n* = 102: 48.3%), whereas they were not confident when selecting and employing proper “INFERENTIAL” statistical tests (*n* = 89: 42.2%), statistical analyses using software (e.g. STATA, SPSS, EpiInfo) (*n* = 85: 40.3%) and drafting a comprehensive plan for data analyses (*n* = 59: 28%) (see Table 3).

**Table 3:** GCC Pharmacists self-perceived competence and confident for planning and conducting health research (*n* = 214)

Research domain	Competent			Confident		
	Competent n (%)	Moderately competent n (%)	Not competent n (%)	Confident n (%)	Moderately competent n (%)	Not confident n (%)
1. Conception of research idea.	91 (42.5)	84 (39.3)	39 (18.2)	101 (47.9)	84 (39.8)	26 (12.3)
2. Searching the literature efficiently.	108 (50.5)	81 (37.9)	25 (11.7)	109 (51.7)	82 (38.9)	20 (9.5)
3. Critically reviewing research literature.	83 (38.8)	93 (43.5)	38 (17.8)	93 (44.1)	87 (41.2)	31 (14.7)
4. Formulating research hypotheses and research questions.	77 (36.0)	95 (44.4)	42 (19.6)	76 (36.0)	101 (47.9)	34 (16.1)
5. Proposing appropriate study designs or methods.	72 (33.6)	86 (40.2)	56 (26.2)	78 (37.0)	92 (43.6)	41 (19.4)
6. Writing research proposal or developing a protocol.	78 (36.4)	89 (41.6)	47 (22.0)	77 (36.5)	89 (42.2)	45 (21.3)
7. Defining target population, sample and eligibility criteria.	90 (42.1)	80 (37.4)	44 (20.6)	77 (36.5)	86 (40.8)	48 (22.7)
8. Determine appropriate sample size.	61 (28.5)	85 (39.7)	68 (31.8)	59 (28.0)	94 (44.5)	58 (27.5)
9. Choosing an appropriate sampling technique (e.g. random sampling).	68 (31.8)	86 (40.2)	60 (28.0)	60 (28.4)	94 (44.5)	57 (27.0)
10. Determining outcome measures (variables to measure).	70 (32.7)	90 (42.1)	54 (25.2)	75 (35.5)	91 (43.1)	45 (21.3)
11. Ethical considerations.	102 (47.7)	77 (36.0)	35 (16.4)	102 (48.3)	72 (34.1)	37 (17.5)
12. Outlining detailed statistical plans to be used in data analyses.	54 (25.6)	85 (40.3)	72 (34.1)	60 (28.4)	92 (43.6)	59 (28.0)
13. Designing a data collection form.	75 (35.5)	89 (42.2)	47 (22.3)	78 (37.0)	87 (41.2)	46 (21.8)
14. Developing and validating a study instrument (e.g. questionnaire).	71 (33.6)	85 (40.3)	55 (26.1)	72 (34.1)	89 (42.2)	50 (23.7)
15. Collecting relevant data using preplanned data collection forms.	79 (37.4)	88 (41.7)	44 (20.9)	91 (43.1)	76 (36.0)	44 (20.9)
16. Managing and storing data including data entry into a database.	84 (39.8)	74 (35.1)	53 (25.1)	81 (38.4)	81 (38.4)	49 (23.2)
17. Statistical analyses using software (e.g. STATA, SPSS, EpiInfo).	56 (26.5)	62 (29.4)	93 (44.1)	51 (24.2)	75 (35.5)	85 (40.3)
18. Choosing and applying appropriate “INFERENTIAL” statistical tests and methods.	43 (20.4)	69 (32.7)	99 (46.9)	46 (21.8)	76 (36.0)	89 (42.2)
19. Summarizing data in tables or charts.	94 (44.5)	78 (37.0)	39 (18.5)	97 (46.0)	79 (37.4)	35 (16.6)
20. Interpretation of the findings and determining the significance of obtained results.	80 (37.9)	83 (39.3)	48 (22.7)	93 (44.1)	77 (36.5)	41 (19.4)
21. Preparing a presentation (oral or poster).	126 (59.7)	61 (28.9)	24 (11.4)	118 (55.9)	73 (34.6)	20 (9.5)
22. Writing a manuscript for publication in a scientific journal.	76 (36.0)	77 (36.5)	58 (27.5)	80 (37.9)	81 (38.4)	50 (23.7)

Comparison of pharmacists’ (*n* = 211) self-perceived level of competence and confidence when planning and conducting PPBR was done among each GCC country separately, and data showed that there were no significant differences (*p* > 0.05). However, there were significant differences between the individual GCC countries in the competent domain, specifically when determining outcome measures (variables to measure) (*p* = 0.030), designing a data collection form (*p* = 0.035) and preparing a presentation (*p* = 0.013) (see Table 4).

**Table 4:** Comparison of pharmacists self-perceived level of competent and confidence for planning and conducting PPBR among each individual GCC country separately (*n* = 211)

Research domain	GCC Countries	Competent			*p*-value	Confident			*p*-value
		Competent	Moderately Competent	Not competent		Confident	Moderately Confident	Not confident	
**1. Conception of research idea.**	KSA	39 (43.3)	33 (36.7)	18 (20.0)	0.806	46 (51.1)	31 (34.4)	13 (14.4)	0.691
	UAE	12 (40.0)	14 (46.7)	4 (13.3)		14 (46.7)	11 (36.7)	5 (16.7)	
	Kuwait	14 (35.0)	17 (42.5)	9 (22.5)		18 (45.0)	17 (42.5)	5 (12.5)	
	Bahrain	7 (63.3)	3 (27.3)	1 (9.1)		6 (54.5)	4 (36.4)	1 (9.1)	
	Oman	19 (47.5)	14 (35.0)	7 (17.5)		17 (42.5)	21 (52.5)	2 (5.0)	
**2. Searching the literature efficiently.**	KSA	43 (47.8)	36 (40.0)	11 (12.2)	0.885	40 (44.4)	40 (44.4)	10 (11.1)	0.744
	UAE	15 (50.0)	13 (43.3)	2 (6.7)		17 (56.7)	9 (30.0)	4 (13.3)	
	Kuwait	20 (50.0)	15 (37.5)	5 (12.5)		22 (55.0)	16 (40.0)	2 (5.0)	
	Bahrain	7 (63.3)	3 (27.3)	1 (9.1)		6 (54.5)	4 (36.4)	1 (9.1)	
	Oman	23 (57.5)	11 (27.5)	6 (15.0)		24 (60.0)	13 (32.5)	3 (7.5)	
**3. Critically reviewing research literature.**	KSA	33 (36.7)	42 (46.7)	15 (16.7)	0.387	36 (40.0)	35 (38.9)	19 (21.1)	0.633
	UAE	12 (40.0)	16 (53.3)	2 (6.7)		15 (50.0)	12 (40.0)	3 (10.0)	
	Kuwait	14 (35.0)	17 (42.5)	9 (22.5)		18 (45.0)	17 (42.5)	5 (12.5)	
	Bahrain	6 (54.5)	2 (18.2)	3 (27.3)		6 (54.5)	4 (36.4)	1 (9.1)	
	Oman	18 (45.0)	13 (32.5)	9 (22.5)		18 (45.0)	19 (47.5)	3 (7.5)	
**4. Formulating research hypotheses and research questions.**	KSA	35 (38.9)	37 (41.1)	18 (20.0)	0.929	33 (36.7)	41 (45.6)	16 (17.8)	0.957
	UAE	11 (36.7)	15 (50.0)	4 (13.3)		12 (40.0)	13 (43.3)	5 (16.7)	
	Kuwait	11 (27.5)	19 (47.5)	10 (25.0)		12 (30.0)	23 (57.5)	5 (12.5)	
	Bahrain	5 (45.5)	4 (36.4)	2 (18.2)		3 (27.3)	6 (54.5)	2 (18.2)	
	Oman	15 (37.5)	17 (42.5)	8 (20.0)		16 (40.0)	18 (45.0)	6 (15.0)	
**5. Proposing appropriate study designs or methods.**	KSA	33 (36.7)	30 (33.3)	27 (30.0)	0.309	32 (35.6)	39 (43.3)	19 (21.1)	0.742
	UAE	10 (33.3)	14 (46.7)	6 (20.0)		12 (40.0)	13 (43.3)	5 (16.7)	
	Kuwait	8 (20.0)	20 (50.0)	12 (30.0)		11 (27.5)	18 (45.0)	11 (27.5)	
	Bahrain	6 (54.5)	2 (18.2)	3 (27.3)		5 (45.5)	5 (45.5)	1 (9.1)	
	Oman	15 (37.5)	17 (42.5)	8 (20.0)		18 (45.0)	17 (42.5)	5 (12.5)	
**6. Writing research proposal or developing a protocol.**	KSA	34 (37.8)	34 (37.8)	22 (24.4)	0.224	32 (35.6)	35 (38.9)	23 (25.6)	0.359
	UAE	11 (36.7)	11 (36.7)	8 (26.7)		13 (43.3)	11 (36.7)	6 (20.0)	
	Kuwait	8 (20.0)	21 (52.5)	11 (27.5)		9 (22.5)	21 (52.5)	10 (25.0)	
	Bahrain	5 (45.5)	4 (36.4)	2 (18.2)		4 (36.4)	5 (45.5)	2 (18.2)	
	Oman	20 (50.0)	16 (40.0)	4 (10.0)		19 (47.5)	17 (42.5)	4 (10.0)	
**7. Defining target population, sample and eligibility criteria.**	KSA	37 (41.1)	33 (36.7)	20 (22.2)	0.925	31 (34.4)	36 (40.0)	23 (25.6)	0.472
	UAE	14 (46.7)	10 (33.3)	6 (20.0)		11 (36.7)	11 (36.7)	8 (26.7)	
	Kuwait	15 (37.5)	16 (40.0)	9 (22.5)		14 (35.0)	15 (37.5)	11 (27.5)	
	Bahrain	6 (54.5)	2 (18.2)	3 (27.3)		5 (45.5)	3 (27.3)	3 (27.3)	
	Oman	18 (45.0)	16 (40.0)	6 (15.0)		16 (40.0)	21 (52.5)	3 (7.5)	
**8. Determine appropriate sample size.**	KSA	24 (26.7)	34 (37.8)	32 (35.6)	0.659	19 (21.1)	43 (47.8)	28 (31.1)	0.167
	UAE	12 (40.0)	10 (33.3)	8 (26.7)		13 (43.3)	9 (30.0)	8 (26.7)	
	Kuwait	9 (22.5)	15 (37.5)	16 (40.0)		9 (22.5)	17 (42.5)	14 (35.0)	
	Bahrain	3 (27.3)	6 (54.5)	2 (18.2)		3 (27.3)	6 (54.5)	2 (18.2)	
	Oman	13 (32.5)	17 (42.5)	10 (25.0)		15 (37.5)	19 (47.5)	6 (15.0)	
**9. Choosing an appropriate sampling technique (e.g. random sampling).**	KSA	25 (27.8)	37 (41.1)	28 (31.1)	0.912	22 (24.4)	44 (48.9)	24 (26.7)	0.456
	UAE	11 (36.7)	12 (40.0)	7 (23.3)		8 (26.7)	14 (46.7)	8 (26.7)	
	Kuwait	11 (27.5)	16 (40.0)	13 (32.5)		9 (22.5)	17 (42.5)	14 (35.0)	
	Bahrain	5 (45.5)	4 (36.4)	2 (18.2)		3 (27.3)	5 (45.5)	3 (27.3)	
	Oman	15 (37.5)	15 (37.5)	10 (25.0)		18 (45.0)	14 (35.0)	8 (20.0)	
**10. Determining outcome measures (variables to measure).**	KSA	33 (36.7)	30 (33.3)	27 (30.0)	0.030*	31 (34.4)	40 (44.4)	19 (21.1)	0.955
	UAE	10 (33.3)	13 (43.3)	7 (23.3)		12 (40.0)	12 (40.0)	6 (20.0)	
	Kuwait	6 (15.0)	19 (47.5)	15 (37.5)		11 (27.5)	19 (47.5)	10 (25.0)	
	Bahrain	5 (45.5)	4 (36.4)	2 (18.2)		4 (36.4)	4 (36.4)	3 (27.3)	
	Oman	15 (37.5)	22 (55.0)	3 (7.5)		17 (42.5)	16 (40.0)	7 (17.5)	
**11. Ethical considerations.**	KSA	45 (50.0)	28 (31.1)	17 (18.9)	0.823	44 (48.9)	22 (24.4)	24 (26.7)	0.089
	UAE	12 (40.0)	14 (46.7)	4 (13.3)		14 (46.7)	13 (43.3)	3 (10.0)	
	Kuwait	16 (40.0)	17 (42.5)	7 (17.5)		18 (45.0)	16 (40.0)	6 (15.0)	
	Bahrain	6 (54.5)	3 (27.3)	2 (18.2)		7 (63.6)	3 (27.3)	1 (9.1)	
	Oman	21 (52.5)	14 (35.0)	5 (12.5)		19 (47.5)	18 (45.0)	3 (7.5)	
**12. Outlining detailed statistical plans to be used in data analyses.**	KSA	20 (22.2)	38 (42.2)	32 (35.6)	0.393	20 (22.2)	44 (48.9)	26 (28.9	0.195
	UAE	8 (26.7)	11 (36.7)	11 (36.7)		11 (36.7)	13 (43.3)	6 (20.0)	
	Kuwait	7 (17.5)	15 (37.5)	18 (45.0)		10 (25.0)	13 (32.5)	17 (42.5)	
	Bahrain	4 (36.4)	4 (36.4)	3 (27.3)		3 (27.3)	6 (54.5)	2 (18.2)	
	Oman	15 (37.5)	17 (42.5)	8 (20.0)		16 (40.0)	16 (40.0)	8 (20.0)	
**13. Designing a data collection form.**	KSA	30 (33.3)	35 (38.9)	25 (27.8)	0.035*	31 (34.4)	39 (43.3)	20 (22.2)	0.472
	UAE	11 (36.7)	12 (40.0)	7 (23.3)		12 (40.0)	13 (43.3)	5 (16.7)	
	Kuwait	13 (32.5)	16 (40.0)	11 (27.5)		13 (32.5)	15 (37.5)	12 (30.0)	
	Bahrain	4 (36.4)	6 (54.5)	1 (9.1)		2 (18.2)	7 (63.6)	2 (18.2)	
	Oman	17 (42.5)	20 (50.0)	3 (7.5)		20 (50.0)	13 (32.5)	7 (17.5)	
**14. Developing and validating a study instrument (e.g. questionnaire).**	KSA	33 (36.7)	34 (37.8)	23 (25.6)	0.421	29 (32.2)	37 (41.1)	24 (26.7)	0.859
	UAE	10 (33.3)	13 (43.3)	7 (23.3)		12 (40.0)	13 (43.3)	5 (16.7)	
	Kuwait	12 (30.0)	12 (30.0)	16 (40.0)		12 (30.0)	16 (40.0)	12 (30.0)	
	Bahrain	4 (36.4)	6 (54.5)	1 (9.1)		3 (27.3)	6 (54.5)	2 (18.2)	
	Oman	12 (30.0)	20 (50.0)	8 (20.0)		16 (40.0)	17 (42.5)	7 (17.5)	
**15. Collecting relevant data using preplanned data collection forms.**	KSA	33 (36.7)	40 (44.4)	17 (18.9)	0.745	40 (44.4)	29 (32.2)	21 (23.3)	0.823
	UAE	9 (30.0)	13 (43.3)	8 (26.7)		14 (46.7)	11 (36.7)	5 (16.7)	
	Kuwait	14 (35.0)	16 (40.0)	10 (25.0)		13 (32.5)	18 (45.0)	9 (22.5)	
	Bahrain	3 (27.3)	6 (54.5)	2 (18.2)		4 (36.4)	4 (36.4)	3 (27.3)	
	Oman	20 (50.0)	13 (32.5)	7 (15.7)		20 (50.0)	14 (35.0)	6 (15.0)	
**16. Managing and storing data including data entry into a database.**	KSA	37 (41.1)	29 (32.2)	24 (26.7)	0.466	35 (38.9)	32 (35.6)	23 (25.6)	0.332
	UAE	10 (33.3)	14 (46.7)	6 (20.0)		15 (50.0)	11 (36.7)	4 (13.3)	
	Kuwait	14 (35.0)	14 (35.0)	12 (30.0)		12 (30.0)	18 (45.0)	10 (25.0)	
	Bahrain	2 (18.2)	5 (45.5)	4 (36.4)		1 (9.1)	7 (63.6)	3 (27.3)	
	Oman	21 (52.5)	12 (30.0)	7 (17.5)		18 (45.0)	13 (32.5)	9 (22.5)	
**17. Statistical analyses using software (e.g. STATA, SPSS, EpiInfo).**	KSA	24 (26.7)	22 (24.4)	44 (48.9)	0.439	21 (23.3)	26 (28.9)	43 (47.8)	0.492
	UAE	7 (23.3)	12 (40.0)	11 (36.7)		9 (30.0)	13 (43.3)	8 (26.7)	
	Kuwait	7 (17.5)	16 (40.0)	17 (42.5)		8 (20.0)	15 (37.5)	17 (42.5)	
	Bahrain	4 (36.4)	2 (18.2)	5 (45.5)		3 (27.3)	3 (27.3)	5 (45.5)	
	Oman	14 (35.0)	10 (25.0)	16 (40.0)		10 (25.0)	18 (45.0)	12 (30.0)	
**18. Choosing and applying appropriate “INFERENTIAL” statistical tests and methods.**	KSA	20 (22.2)	26 (28.9)	44 (48.9)	0.407	21 (23.3)	28 (31.1)	41 (45.6)	0.131
	UAE	7 (23.3)	12 (40.0)	11 (36.7)		9 (30.0)	12 (40.0)	9 (30.0)	
	Kuwait	3 (7.5)	14 (35.0)	23 (57.5)		3 (7.5)	15 (37.5)	22 (55.0)	
	Bahrain	2 (18.2)	5 (45.5)	4 (36.4)		1 (9.1)	6 (54.5)	4 (36.4)	
	Oman	11 (27.5)	12 (30.0)	17 (42.5)		12 (30.0)	15 (37.5)	13 (32.5)	
**19. Summarizing data in tables or charts.**	KSA	40 (44.4)	34 (37.8)	16 (17.8)	0.969	38 (42.2)	39 (43.3)	13 (14.4)	0.734
	UAE	12 (40.0)	13 (43.3)	5 (16.7)		15 (50.0)	10 (33.3)	5 (16.7)	
	Kuwait	17 (42.5)	15 (37.5)	8 (20.0)		17 (42.5)	14 (35.0)	9 (22.5)	
	Bahrain	4 (36.4)	4 (36.4)	3 (27.3)		4 (36.4)	5 (45.5)	2 (18.2)	
	Oman	21 (52.5)	12 (30.0)	7 (17.5)		23 (57.5)	11 (27.5)	6 (15.0)	
**20. Interpretation of the findings and determining the significance of obtained results.**	KSA	32 (35.6)	33 (36.7)	25 (27.8)	0.925	39 (43.3)	32 (35.6)	19 (21.1)	0.976
	UAE	11 (36.7)	14 (46.7)	5 (16.7)		14 (46.7)	9 (30.0)	7 (23.3)	
	Kuwait	15 (37.5)	16 (40.0)	9 (22.5)		17 (42.5)	17 (42.5)	6 (15.0)	
	Bahrain	5 (45.5)	4 (36.4)	2 (18.2)		4 (36.4)	5 (45.5)	2 (18.2)	
	Oman	17 (42.5)	16 (40.0)	7 (17.5)		19 (47.5)	14 (35.0)	7 (17.5)	
**21. Preparing a presentation (oral or poster).**	KSA	49 (54.4)	28 (31.1)	13 (14.4)	0.013*	46 (51.1)	34 (37.8)	10 (11.1)	0.335
	UAE	14 (46.7)	12 (40.0)	4 (13.3)		18 (60.0)	8 (26.7)	4 (13.3)	
	Kuwait	27 (67.5)	11 (27.5)	2 (5.0)		23 (57.5)	16 (40.0)	1 (2.5)	
	Bahrain	7 (63.6)	2 (18.2)	2 (18.2)		4 (36.4)	6 (54.5)	1 (9.1)	
	Oman	29 (72.5)	8 (20.0)	3 (7.5)		27 (67.5)	9 (22.5)	4 (10.0)	
**22. Writing a manuscript for publication in a scientific journal.**	KSA	35 (38.9)	28 (31.1)	27 (30.0)	0.613	35 (38.9)	31 (34.4)	24 (26.7)	0.594
	UAE	13 (43.3)	12 (40.0)	5 (16.7)		12 (40.0)	13 (43.3)	5 (16.7)	
	Kuwait	11 (27.5)	15 (37.5)	14 (35.0)		11 (27.5)	19 (47.5)	10 (25.0)	
	Bahrain	3 (27.3)	6 (54.5)	2 (18.2)		3 (27.3)	6 (54.5)	2 (18.2)	
	Oman	14 (35.0)	16 (40.0)	10 (25.0)		19 (47.5)	12 (30.0)	9 (22.5)	

## Discussion

As far as we are aware this is the first study to examine the research background of the GCC pharmacists and determine their barriers to conducting PPBR. In addition, it is believed that this study is unique in determining the self-perceived level of competence and confidence when planning and conducting PPBR among pharmacists in GCC countries all together and separately.

GCC pharmacists’ proportion with prior research experience as acknowledged by participants (70%) was higher than those reported in past literature, which ranged from 25 to 59% [6, 9, 10, 19, 20]. However, this proportion was lower than the proportion of Nigerian pharmacists (79.5%) in Abubakar et al.’s study in 2018. This high proportion could be justified by the high number of research graduation projects conducted by undergraduates in the Gulf, which is a mandatory requirement in order to earn a bachelor’s degree.

Nearly three-quarters of the sample of the GCC pharmacists (72%) had received training courses such as workshops or seminars, during their undergraduate studies or in the workplace, which was in line with the finding in two previous studies conducted in Qatar [19, 20].

In this study, volunteering as a participant in a study (15%) or being a principal investigator or co-investigator (8%) were the most reported research activities by participants, which was in line with Zeidan et al.’s (2019) study, who reported that only 8% of Lebanese pharmacists were either principal investigators or co-investigators as well as data collectors, when they were involved in research previously. One of the self-reported research activities is publication rate, which was found to be low in this study. The low publication rate reported in this study was consistent with Abubakar et al.’s study (2018) in Nigeria and Awaisu et al.’s study (2015) in Qatar. Low self-esteem, humble mentoring, the absence of time and innovation were the reasons that mainly contributed to the low publication rate, as reported by a single study carried out by Vouri et al. in 2015 [21]. Despite the high level of interest in conducting PPBR reported in this study, which was consistent with past studies [7, 10, 11, 15], this great interest does not translate into high research productivity. This could be due to barriers found in the work area, as reported by participants. Lack of time was the most acknowledged barrier as reported by this study’s participants, followed by the absence of the following: sufficient knowledge, financial and job support. As reported by previous studies, time constraints were the most common challenge in conducting PPBR [7, 11, 12]. Lack of adequate knowledge, job support and funds were also other reported barriers in previous studies [5, 7, 9–12, 15, 20]. In order for PPBR to continue to evolve in response to changing health care needs, pharmacists must be encourage to conduct more research. In addition, pharmacists should contribute in research ideas and be involved in the research design, data analysis or report writing not only collect data or being a participant. In addition, all reported barriers should be recognized and resolved. For example, in terms of time, financial and job support, provision for paying adequate compensation for the time spent on research either in the form of relief staff or financially is important. Researchers can also help alleviate this burden by being very mindful of time constraints when designing research protocols and paperwork; consulting with practitioners at this stage of planning would be prudent. Moreover, a research assistant can be suggested to provide relief and to encourage a supportive working environment for pharmacy practice researchers.

Contradictory to earlier findings [7, 12, 15] which showed that pharmacists lack confidence when undertaking PPBR, this study revealed that GCC pharmacists’ self-competence and confidence levels to undertake PPBR were high, which was in line with Elkassem et al.’s (2013) study in Qatar and Sultana et al.’s (2016) in KSA. In addition, GCC pharmacists in this study revealed that they had adequate training and skills and rated their abilities as good to excellent when planning and undertaking PPBR. In this study, there was a significant difference among participants with previous research experience (K-W: *p* = 0.001) and previous research training (K-W: *p* = 0.003) and their views on their overall ability to design and conduct PPBR. Participants with previous research experience and training were found to be more able to plan and conduct PPBR. These finding are supported by two past studies which revealed that students’ exposure to research projects during their undergraduate studies was associated with more positive attitudes and greater confidence when planning and conducting PPBR [20, 22]. However, in this study GCC pharmacists were found to be neither competent nor confident in conducting research statistics and determining the right sample size, which was consistent with three previously published studies [5, 11, 15]. When comparing self-perceived level of competence and confidence among GGC pharmacists for each individual country, data showed that there were no significant differences between these countries (*p* > 0.05).

The Pharmacy Practice Research Network (PPRN) and continuous training of GCC pharmacists were seen to be efficient strategies used to tackle low competence and confidence among pharmacists [23, 24], by providing a means for clinical pharmacists with common practice and research interests together for professional interaction, networking, and continuing education. However, activities may vary depending on the interests and perceived needs of their members. A previous study of pharmacy students by Perez et al. (2017) reported that pharmacy students’ research training programs increased their confidence to conduct tasks that were related to research and also increased their rate of publication [25]. However, research courses and training provided for pharmacists in the Gulf is still poor. In Saudi Arabia for example, there are around twenty eight universities that grant pharmacy degrees, some offering a Bachelor in Pharmacy (BPharm), whereas most pharmacy colleges have moved to a Doctor of Pharmacy degree (PharmD) which integrates more pharmacy practice and clinical pharmacy courses within the curriculum rather than research training courses [26]. In addition, PharmD programme also eliminates some of the extra basic pharmaceutical sciences courses, such as pharmacognosy, medicinal chemistry, biostatistics, pharmacoepidemiology and research methodology [26]. Therefore, there is a need for research-oriented training and these training programmes should mainly focus on biostatistics and data analysis based on the interests and perceived needs of pharmacy practice researchers. Providing the needed skills or the acquired knowledge is very important for these researchers especially in regards to conducting research statistics and determining the right sample size which could be due to the lack of expertise or training on how to utilise any software or the required tests for analysis. Therefore, this type of training should be integral to university curriculum. The Ministry of Education should take the initiative in revising the current pharmacy curriculum to include courses on biostatistics, pharmacoepidemiology and research methodology. Moreover, providing organisational and administrative support on these aspects throughout the years of study might be useful and perhaps by putting pharmacists with less research experience in contact with those who are more experienced.

### Limitations

Although participants were collected during SIPHA and DUPHAT annual conferences, which gathers delegate from all GCC countries, findings of this study may not be generalizable because of the convenience sampling technique applied, which could be vulnerable to selection bias and sampling errors. Another limitation is that the subjective assessment of competence and confidence is liable to self-reporting bias, in addition to recall bias. In addition, the research domains included in the questionnaires were considered as serial dependent questions. Thus, this design did not assist in generating compelling results on pharmacists’ overall research competency which was one of the limitations of this study.

## Conclusions

This study’s findings support the fact that GGC pharmacists realize the importance and value of planning and conducting PPBR for their practice and are more confident and competent to undertake and contribute to research, compared to pharmacists from other countries. However, being interested in research does not correspond to GCC pharmacists’ actual ability to plan or undertake PPBR. Pharmacists’ self-reported competence and confidence to undertake statistical tests of data and determine sample size were weak. Addressing the weaknesses and barriers reported by GGC pharmacists may promote the implementation of research outcomes in the Gulf countries.

## Data Availability

The data file can be made available on request to the authors.

## Abbreviations

PPBR: Pharmacy practice-based research
GCC: The Gulf Cooperation Council
SIPHA: Pharmaceutical Sciences Annual National Conference
DUPHAT: Dubai International Pharmaceuticals and Technologies Conference and Exhibition
WHO: The World Health Organisation
FIP: The International Pharmaceutical Federation
QR: A quick response code
K-W: Kruskal-Wallis test
MWU: Mann-Whitney-U

## References

[1] Mays N (1997). A new age for pharmacy practice research: promoting evidence-based practice in pharmacy.

[2] Awaisu A, Alsalimy N (2015). Pharmacists’ involvement in and attitudes toward pharmacy practice research: a systematic review of the literature. Res Soc Adm Pharm.

[3] Kuipersa E, Wensinga M, De Smet P, Teichert M (2019). Barriers and facilitators for community pharmacists’ participation in pharmacy practice research: a survey. Int J Pharm Pract.

[4] Habeeb IAR, Jose D, Jegan RS (2012). Pharmacists in the wider public health workforce – a review. Arch Pharm Pract.

[5] Adisa R, Olukotun RT, Morawo PK, Fakeye TO (2017). Hospital and community pharmacists’ perception of the scope, barriers and challenges of pharmacy practice-based research in Nigeria. J Pharm Pract.

[6] Sam AT, Parasuraman S (2015). The nine-star pharmacist: an overview. J Young Pharm.

[7] Awaisu A, Bakdach D, Elajez RH, Zaidan M (2015). Hospital pharmacists’ self-evaluation of their competence and confidence in conducting pharmacy practice research. Saudi Pharm J.

[8] Deal EN, Stranges PM, Maxwell WD, Bacci J, Ashjian EJ, DeRemer DL (2016). The importance of research and scholarly activity in pharmacy training. Pharmacotherapy..

[9] Sultana K, Al Jeraisy M, Al Ammari M, Patel R, Zaidi ST (2016). Attitude, barriers and facilitators to practice-based research: cross-sectional survey of hospital pharmacists in Saudi Arabia. J Pharm Policy Pract.

[10] Peterson GM, Jackson SL, Fitzmaurice KD, Gee PR (2009). Attitudes of Australian pharmacists towards practice-based research. J Clin Pharm Ther.

[11] Perreault MM, Thiboutot Z, Burry LD, Rose L, Kanji S, LeBlanc JM (2012). Canadian survey of critical care pharmacists’ views and involvement in clinical research. Ann Pharmacother.

[12] Armour C, Brillant M, Krass I (2007). Pharmacists’ views on involvement in pharmacy practice research: strategies for facilitating participation. J Pharm Pract.

[13] Hebert J, Laliberté MC, Berbiche D, Martin E, Lalonde L (2013). The willingness of community pharmacists to participate in a practice-based research network. Can Pharm J.

[14] Stranges PM, Vouri SM, Bergfeld F, Crain M, Crain M, Jindal N (2015). Pharmacy resident publication success: factors of success based on abstracts from a regional meeting. Curr Pharm Teach Learn.

[15] Abubakar U, Sulaiman SA, Usman MN, Umar MD (2018). Nigerian pharmacists’ self-perceived competence and confidence to plan and conduct pharmacy practice research. J Pharm Pract.

[16] GMP event (2020). DUPHAT 2020.

[17] Sampsize.sourceforge.net. (2020). Sampsize [Online]. Available at: http://sampsizesourceforgenet/iface/ Accessed 04 April 2020.

[18] Zhang Y, Hedo R, Rivera A, Richardson S, Tu XM (2019). Post hoc power analysis: is it an informative and meaningful analysis?. BMJ..

[19] Elkassem W, Pallivalapila A, Al Hail M, McHattie L, Diack L, Stewart D (2013). Advancing the pharmacy practice research agenda: views and experiences of pharmacists in Qatar. Int J Clin Pharm.

[20] Zeidan RK, Hallit S, Zeenny RM, Salameh P (2019). Lebanese community-based pharmacists’ interest, practice, knowledge, and barriers towards pharmacy practice research: a cross-sectional study. Saudi Pharm J.

[21] Vouri SM, Stranges PM, Burke JM, Micek S, Pitlick MK, Wenger P (2015). The importance of research during pharmacy residency training. Curr Pharm Teach Learn.

[22] Kritikos VS, Saini B, Carter S, Salameh P (2015). Factors influencing pharmacy students’ attitudes towards pharmacy practice research and strategies for promoting research interest in pharmacy practice. Pharm Pract (Granada).

[23] Koster ES, Rump W, Blom L, Rump W, Bouvy ML (2014). The Utrecht pharmacy practice network for education and research: a network of community and hospital pharmacies in the Netherlands. Int J Clin Pharm.

[24] Awaisu A, Kheir N, Alrowashdeh HA, Allouch SN, Jebara T, Zaidan M (2015). Impact of a pharmacy practice research capacity-building programme on improving the research abilities of pharmacists at two specialised tertiary care hospitals in Qatar: a preliminary study. J Pharm Health Serv Res.

[25] Perez A, Rabionet MS, Bleidt B (2017). Teaching research skills to student pharmacists in one semester: an applied research elective. Am J Pharm Educ.

[26] Al-jedai A, Qaisi S, Al-meman A (2016). Pharmacy practice and the healthcare system in Saudi Arabia. Int Perspect Pharm Pract J.

